# Comparative Genomics Reveals Potential Mechanisms of Plant Beneficial Effects of a Novel Bamboo-Endophytic Bacterial Isolate *Paraburkholderia sacchari* Suichang626

**DOI:** 10.3389/fmicb.2021.686998

**Published:** 2021-06-18

**Authors:** Kai Wang, Ying Wu, Mengyuan Ye, Yifan Yang, Fred O. Asiegbu, Kirk Overmyer, Shenkui Liu, Fuqiang Cui

**Affiliations:** ^1^State Key Laboratory of Subtropical Silviculture, Zhejiang A&F University, Hangzhou, China; ^2^Faculty of Agriculture and Forestry, University of Helsinki, Helsinki, Finland; ^3^Organismal and Evolutionary Biology Research Program, Faculty of Biological and Environmental Sciences, and Viikki Plant Science Centre, University of Helsinki, Helsinki, Finland

**Keywords:** bamboo, *Paraburkholderia*, growth-promoting bacteria, comparative genomics, genome

## Abstract

Plant-beneficial microbes have drawn wide attention due to their potential application as bio-control agents and bio-fertilizers. Moso bamboo, which is among the monocots with the highest growth rate, lives perennially with abundant microbes that may benefit annually growing crops. Genome information of moso bamboo associated bacteria remains underexplored. We isolated and identified a novel *Paraburkholderia* strain Suichang626 from moso bamboo roots. Growth promoting effects of Suichang626 on both moso bamboo and seedlings of the model dicot *Arabidopsis thaliana* were documented in laboratory conditions. To gain insight into the genetic basis of this growth promotion effect, we sequenced the genome of Suichang626. Evidenced by genome-wide phylogeny data, we propose that Suichang626 is a novel strain of *Paraburkholderia sacchari*. Gene homologs encoding biosynthesis of the plant growth-promoting chemicals, acetoin and 2,3-butanediol, were identified in the genome of Suichang626. Comparative genomics was further performed with plant-beneficial and plant/animal pathogenic species of *Paraburkholderia* and *Burkholderia*. Genes related to volatile organic compounds, nitrogen fixation, and auxin biosynthesis were discovered specifically in the plant growth-promoting species of both genera.

## Introduction

Moso bamboo (*Phyllostachys edulis*) is widely distributed in southern China. The characteristics of stress tolerance, rapid growth, and high quality fibers make moso bamboo an ideal plant both as a carbon sink and for industrial uses (Peng et al., 2013; Huang and Young, 2019; Churkina et al., 2020; Tao et al., 2020; Ye et al., 2020). Bamboo is one of the largest monocots. Due to its perennial life style, bamboo is considered to harbor more microbes compared to common crops, which are typically annual monocots (Han et al., 2009; Singh et al., 2020). Although some plant growth-promoting (PGP) microbes have been isolated from bamboo (Zhang et al., 2018a, b), their genome sequences remain largely uncharacterized. Previously, we observed the rare phenomenon of enhanced shoot formation from a bamboo rhizome. Specifically, 18 shoots developed within approximately 1 m of rhizome, a space that would normally bear only one or two shoots. With the working hypothesis that PGP microbes are involved in the development of excessive shoots we have previously isolated PGP microbes specifically associated with the rhizosphere of the rhizome exhibiting enhanced shoot production, including several *Paraburkholderia* strains (Cui et al., 2021).

The bacterial genus *Burkholderia* consists of a large group of multifunctional bacteria. The genus *Paraburkholderia* was recently created in order to accommodate the agriculturally beneficial species formerly in the genus *Burkholderia*, which contains species pathogenic to plants, humans, and animals (Sawana et al., 2014; Kaur et al., 2017). Most of the PGP species formerly in *Burkholderia* were redistributed to the genera *Paraburkholderia* and *Caballeronia* (Mannaa et al., 2019). The exact criteria of bifurcation *Paraburkholderia* from *Burkholderia* has been questioned (Eberl and Vandamme, 2016). *Paraburkholderia* strains characteristically lack type III, IV, and VI secretion systems (Kaur et al., 2017), and the GC content of *Paraburkholderia* genomes (61.4–65%) is slightly lower in comparison to *Burkholderia* sensu stricto (65.7–68.5%) (Sawana et al., 2014). Most genomes of PGP *Paraburkholderia* strains contain genes encoding nitrogen fixation machinery and biosynthesis of growth promoting secondary metabolites (Donoso et al., 2017; Kaur et al., 2017; Bernabeu et al., 2018; Hsu et al., 2018). Genome size of *Paraburkholderia* strains varies from around 8–11.5 Mbp (Kaur et al., 2016; Mannaa et al., 2019). Although many of *Paraburkholderia* species have been identified, the genomes of only a few strains are available, none of which were isolated from bamboo (Kaur et al., 2017).

The mechanisms of growth enhancing effects are variable among species and strains within the scope of nutrient supply and PGP metabolite production. Several bacterial genera were capable of nitrogen fixation (Boyd and Peters, 2013; Rosenblueth et al., 2018; Ryu et al., 2020). Bacterial species also produce phytohormones, such as gibberellic acid, auxins, and cytokinins (Spaepen, 2015; Chanclud and Morel, 2016). These microbial derived hormones can directly function as growth stimulators (Kurepin et al., 2014). In addition to direct interaction, the host plant’s own hormone production and homeostasis can be modulated by bacterial enzymes, such as 1-aminocyclopropane-1-carboxylate (ACC) deaminase, which can reduce plant ethylene production (Onofre-Lemus et al., 2009; Kurepin et al., 2014). Bacterial volatile organic compounds (VOCs), such as 2,3-butanediol and acetoin, can promote plant growth, possibly by interacting with plant hormone signaling (Ryu et al., 2003; Bailly and Weisskopf, 2012). A wide variety of bacterial secondary metabolites are also involved in PGP (Sharma and Johri, 2003). The PGP effects of bamboo-associated bacteria have not been explored using bacterial genomics. To gain insight into this process we have isolated and characterized Suichang626, an endophytic *Paraburkholderia* strain associated with a bamboo rhizome that exhibits enhanced shoot production. We demonstrate significant PGP effects on moso bamboo and *Arabidopsis thaliana* seedlings with Suichang626 and utilize genome sequencing and comparative genomic analysis to explore the PGP capabilities of this strain.

## Materials and Methods

### Isolation of *Paraburkholderia sacchari* Suichang626

Roots at the depth of 30–40 cm were collected from the rhizome with enhanced shoot production, which was discovered in Suichang County in southern Zhejiang Province. Samples were stored at 4°C after transport to the laboratory. For isolation, roots were washed with water, surface sterilized with 70% ethanol, cut into pieces, and placed on potato dextrose agar (PDA) medium. The endophytes that propagated on PDA medium were isolated and purified by serial dilution plating. The 16S rRNA region of the top five most effective PGP microbes was sequenced with primers 27F and 1492R (Caporaso et al., 2012). The 16S rRNA region of Suichang626 (MW741820) was queried using BLAST at the NCBI^1^ against a database of 16S ribosomal RNA sequences (Bacteria and Archaea).

### Growth-Promotion Assay

Seeds of the model dicot *Arabidopsis thaliana* (hereafter referred to as *Arabidopsis*) were sterilized with 75% ethanol for 5 min and placed on 1/2x MS medium containing 0.5% sucrose. The PDA-cultivated Suichang626 was streaked on the bottom of the sterile medium without touching the *Arabidopsis* seeds. A plate containing only *Arabidopsis* seeds was used as control. Total 24 seedlings (eight in each replicates) were applied with Suichang626 and another 24 seedlings were used as control. The seedlings on sterile plates were cultivated in a vertical position in a growth chamber (MLR-352H-PC, Panasonic, Japan) with a 12/12 h light/dark cycle at 22°C. Seedlings were photographed and harvested on day 16 and dried at 60°C overnight for determination of whole seedling dry weight. Combined dry weight of three independent replicates were subjected to a statistical analysis with a linear model. The significances were estimated with multcomp package in R (as descripted in Cui et al., 2018). For moso bamboo, seeds were rinsed with tap water overnight and sterilized with 75% ethanol for 30 s. Sterilized seeds were placed on sterile filter paper in a transparent dish at 25°C for 2 days. Germinated seedlings of similar sizes were chosen for the PGP assay. Moso bamboo seedlings were dipped in Suichang626 suspension (OD_600_ = 0.5) and then planted in soil, using uninoculated seedlings as controls. Photographs were taken of 25-day-old seedlings. The aerial parts of each bamboo seedling were dried at 60°C for 2 days and weighed. Total 24 seedlings (eight in each replicates) were analyzed. The same statistical analysis was performed as descripted above for *Arabidopsis*.

### Genome Sequencing and Annotation

The genome of isolate Suichang626 was sequenced with Illumina Hiseq and PacBio methods. For Illumina Hiseq method, the purified genomic DNA was fragmented into 300–500 bp pieces. A single-strand DNA fragment library was built with the TruSeq^TM^ DNA Sample Prep Kit (Illumina)^2^. Single-strand DNA was amplified with TruSeq PE Cluster Kit v3-cBot-HS and sequenced with TruSeq SBS Kit v3- HS for 200 cycles. For PacBio sequencing, high quality genomic DNA was fragmented into 8–10 kb pieces with G-tubes method (Covaris, United States)^3^. Hairpin adaptors were added to both ends of double stranded DNA fragments and SMRTbell structures were created. The genome was assembled from PacBio sequencing data with the canu method (Koren et al., 2017). Genomic rRNA and tRNA were predicted with barrnap and tRNAscan-SE V1.3.1 (Lowe and Chan, 2016). Gene prediction was performed with Glimmer 3.02 (Delcher et al., 2007), after which gene functions were assigned using the best BLAST match (NR database) to predicted genes. Average amino acid identity values among 14 species were determined with the online AAI calculator tool (Rodriguez-R and Konstantinidis, 2016). Digital DNA-DNA hybridization (dDDH) values were calculated with the online GGDC tool (Meier-Kolthoff et al., 2013). Tetranucleotide frequency correlation coefficient was calculated using the JSpecies webtool (Richter et al., 2016).

### Comparative Genomics

The genomes and annotations of 13 *Paraburkholderia* and *Burkholderia* species (assembly accession numbers listed in Table 2) were collected from NCBI. The phylogenetic trees of *Paraburkholderia* and *Burkholderia* species were built with 16S rRNA and genome-wide single-copy protein sequences. 16S rRNA sequences were collected from NCBI or identified from genomes by BLASTn searches. Single-copy proteins from 14 genome annotations were identified using orthofinder with default settings (Emms and Kelly, 2015). For the 16S rRNA phylogeny, multiple sequences were aligned with ClustalX2 (Larkin et al., 2007). Aligned regions of 16S rRNA genes were applied as input sequences in RAxML method (Stamatakis, 2014) with rapid bootstrapping model (1,000×). The genome-wide phylogeny was built as described in Wang et al. (2021). Briefly, alignment quality control of single-copy proteins was monitored by applying sequence scores ≥0.8 in MAFFT analysis with Guidance2 (Sela et al., 2015). Multiple aligned sequences were concatenated using the FASconCAT_V1.0 script. The RAxML method (Stamatakis, 2014) with rapid bootstrapping (100×) model was used for constructing the phylogenetic tree.

Genome alignments between Suichang626 and *P. sacchari*, as well as Suichang626 and *P. bannensis* were performed with Mummer 4.0.0beta2 using nucmer (Marçais et al., 2018). The optimal co-linear order of contigs was shaped with mummerplot with parameter –fat. Mummerplot output.ps files were viewed and edited in CorelDraw. Gene synteny plot of the nitrogen fixation gene cluster was built with the genoPlotR package (Guy et al., 2010) in R. Sequence comparison was done with tBLASTx with these cutoff criteria, *e*-value < e-100 and bit score ≥100. Enzymes (pyruvate decarboxylase, acetolactate synthase, acetoin dehydrogenase, acetoin reductase) involved in the biosynthesis of acetoin and 2,3-butanediol were queried in 13 species. Protein sequences of *Paraburkholderia sacchari* Suichang626 from the genome annotation (swissport) produced in this study were used as references. BLASTp was applied and the candidate enzyme selection criteria were *e*-value < 1e-50, and similarity >40%. The primary query sequences of the auxin [indole acetic acid (IAA)] biosynthesis enzymes were collected from characterized proteins (aromatic-amino-acid aminotransferase: *Ralstonia solanacearum* AOE89064.1, tryptophan 2-monooxygenase: *Pseudomonas savastanoi* P06617.1, tryptophan 2-monooxygenase: *Ralstonia solanacearum* AOE91018.1, indole acetamide hydrolase: *Azoarcus olearius* CAL94572.1, aromatic aminotransferase: *B. mallei* ATCC 10399 EDP86360.1, aromatic-L-amino-acid decarboxylase: *Pseudomonas fluorescens* BBc6R8 ESW55773.1, indole-3-pyruvate decarboxylase: *Cupriavidus necator* H16 CAJ96188.1, and aldehyde dehydrogenase: *Pseudomonas fluorescens* BBc6R8 ESW53875.1). After searching with the primary query, we applied the best hits (similarity >60%, *e*-value < e-100) from *Burkholderia* and *Paraburkholderia* species as secondary query (aldehyde dehydrogenase: *B. ambifaria* WP011658118.1, aromatic aminotransferase: *B. mallei* ATCC 10399 EDP86360.1, indoleacetamide hydrolase: *P. xenovorans* WP011492251.1, alpha-keto acid decarboxylase: *P. xenovorans* WP011493090.1, tryptophan 2,3-dioxygenase: *P. phytofirmans* PsJN ACD17621.1, tryptophan 2-monooxygenase: *P. xenovorans* LB400 ABE37105.1). Results from the BLASTp search (*e*-value < e-70) from the second query were taken account into analysis. The query sequences of known bacterial cytokinin biosynthesis enzymes (isopentenyl transferase: *Pseudomonas savastanoi* pv. savastanoi CBZ39944.1, isopentenyl transferase: *Rhodococcus fascians* D188 YP_007878707.1, cytokinin dehydrogenase/oxidase: *R. fascians* D188 YP_007878708.1, phosphoribohydrolase: *R. fascians* D188 YP_007878709.1, isopentenyl transferase: *Agrobacterium rubi* TR3 = NBRC 13261 GAK72221.1, isopentenyl transferase: *A. tumefaciens* YP_001967412.1, isopentenyl transferase: *A. fabacearum* S56 CUX06613.1, isopentenyl transferase: *Gossypium arboreum* KHG07744.1, cytokinin riboside 5′-monophosphate phosphoribohydrolase LOG family protein: Rhodotorula toruloides NP11 EMS19895.1) were collected, and queried using an *e*-value < 1e-40, similarity >40% for hit criteria. Secondary metabolite gene clusters of 13 species were examined with online tool antiSMASH (Weber et al., 2015).

### Accession Number

This Whole Genome Shotgun project has been deposited at DDBJ/ENA/GenBank under the accession JAGYFL000000000. The version described in this paper is version JAGYFL010000000. Genomic read data were deposited in the Sequence Read Archive (SRA) under the accession number SRR14464902. The biosample accession is SRR14464902. The bioproject accession is PRJNA503302. The 16S rRNA of Suichang636 accession is MW741820.

## Results

### Plant Growth-Promotion Effects of *Paraburkholderia sacchari* Suichang626

**FIGURE 1:**
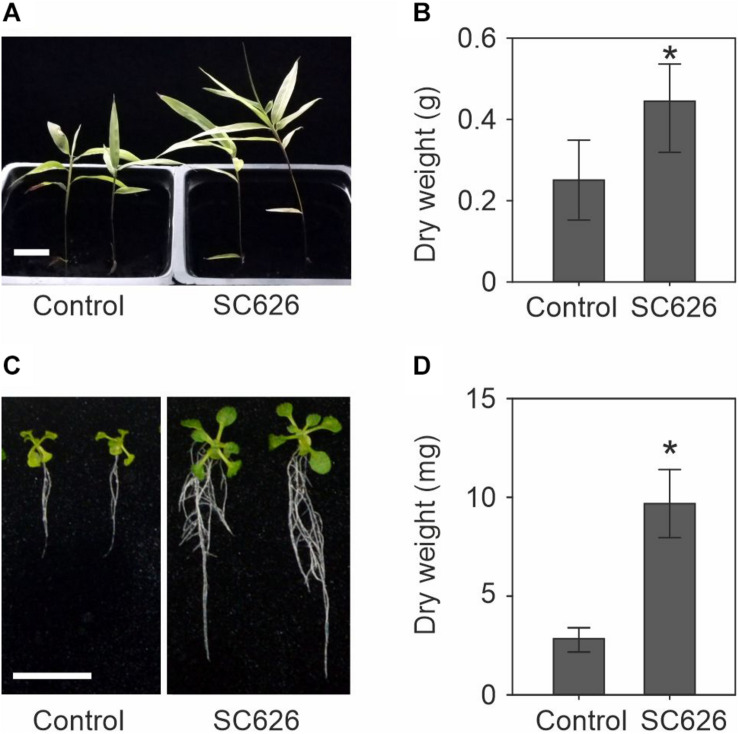
Growth promotion assay of *Paraburkholderia sacchari* Suichang626 (SC626) on seedlings of moso bamboo and *Arabidopsis thaliana*. **(A)** Phenotype of 25-day-old bamboo seedlings in soil with or without *P. sacchari* Suichang626 inoculation. Bar = 1 cm. **(B)** Dry weight of moso bamboo seedlings from **(A)**. **(C)** Symptoms of 16-day-old *Arabidopsis* with or without *P. sacchari* Suichang626 inoculation. Scale bar = 1 cm. **(D)** Quantitative data of the dry weight of seedlings in **(C)**. All experiments were performed three times (*n* = 8 in each biological repeat). *: significant difference (*P* < 0.05).

In Suichang County, Zhejiang Province, China, we have discovered and harvested rhizome material that exhibited greatly enhanced shoot production and used this material to isolate endophytic microbes and screen them for PGP activity. A total of 674 isolates were tested and the 16S rRNA region of the top five most effective PGP microbes was sequenced. Here we present the characterization of bacterial strain Suichang626. The PGP activity was tested with moso bamboo seedling and *Arabidopsis* seedlings. Suichang626 promoted the growth of bamboo seedlings both in vertical and in horizontal direction when compared to control seedlings (Figure 1A). Nearly two-fold enhanced biomass production was gained in seedlings inoculated with Suichang6 according to the dry weight assay (Figure 1B). To investigate PGP activity in other plants, the model dicot plant *Arabidopsis* was assayed. Both the size and biomass of *Arabidopsis* seedlings increased significantly upon co-cultivation with Suichang626 (Figures 1C,D).

### Genome Description of *Paraburkholderia sacchari* Suichang626

The sequenced genome of Suichang626 was assembled into five contigs. The predicted genome size was 8.1 Mb with 64.1% GC content, which are consistent with the genome characteristics of *Paraburkholderia* strains (Kaur et al., 2016; Mannaa et al., 2019). In total 5,300 genes were predicted and annotated (Table 1). Seven copies each of the 16S rRNA and 23S rRNA were found in the Suichang626 genome. Additionally, 1,605 simple repeats and 48 small RNAs were detected (Supplementary File 1).

**TABLE 1:** Genome information of *Paraburkholderia sacchari* Suichang626.

Strain name		*Paraburkholderia sacchari* Suichang626

**Isolation source**		**Moso bamboo root**

**Isolation location**		**Suichang, Zhejiang, China**
Pacbio raw sequence	Total reads number	99597
	Total bases	699.4 Mb
	Average length	7022 bp
	N50	10047
Genome assembly	Contigs number	5
	Total length	8.1 Mb
	GC content	64.1%
	Largest contig length	3.19 Mb
	N50	2198980
	L50	2
	rRNA number	14
	tRNA number	71
Genome annotation	Genes number	5300
	Genes total length	5.04 Mb
	Genes density	0.654 per kb
	Genes average length	951 bp
	Genes/Genome	62.30%

### Suichang626 Is a Strain of *Paraburkholderia sacchari*

**FIGURE 2:**
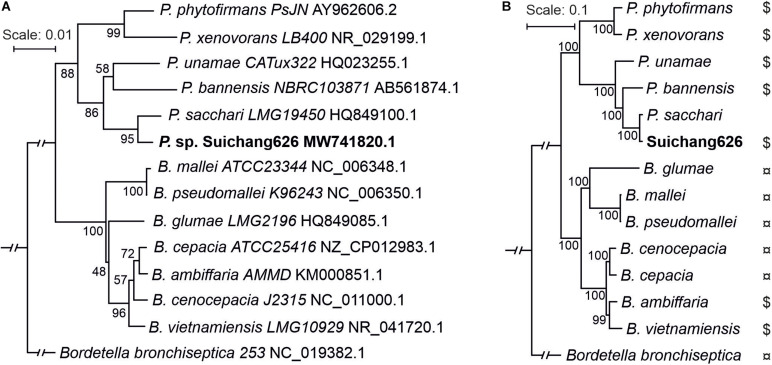
Phylogenetic trees of representative species from genus *Paraburkholderia* and *Burkholderia* using 16S rRNA sequence and genome-wide single-copy protein sequences. **(A)** The 16S rRNA sequences were collected from NCBI or searched from genomes by BLASTn. *Bordetella bronchiseptica* was selected as outgroup. Sequence alignment was performed using ClustalX2 and aligned regions were applied as input for RAxML tree building. Rapid bootstrapping model (1,000×) was chosen. Bootstrap percentage values are indicated at each node. **(B)** Phylogenetic tree constructed by using 795 single-copy protein sequences. Protein sequences were selected from genome annotations. Single-copy proteins were produced with orthofinder (default settings). Sequence alignment quality control was achieved by applying sequence scores ≥0.8 in MAFFT analysis with Guidance2. Multiple aligned sequences were gathered into one sequence using FASconCAT_V1.0. RAxML and rapid bootstrapping (100×) were used for constructing phylogenetic tree. Bootstrap values are indicated at each node. $, plant beneficial bacteria; ¤, plant/animal pathogen.

BLAST searches revealed that the Suichang626 16S rRNA region was 99% identical to *Paraburkholderia sacchari* LMG19450, indicating that Suichang626 belongs to the genus *Paraburkholderia* and suggesting it may be a strain of *P. sacchari*. In order to define the phylogenetic position of strain Suichang626, we performed phylogenetic analyses with 16S rRNA and genome-wide single-copy protein sequences. Both phylogenetic trees revealed that Suichang626 was most closely related to *P. sacchari* LMG19450 (Figure 2), further suggesting that the Suichang626 isolate is a strain of the species *P. sacchari*. A genome-wide maximum-likelihood phylogenetic tree was constructed across the genera *Paraburkholderia* and *Burkholderia*, using 795 single-copy protein sequences with several representative species of each genus included. The genome-wide tree separated the genera *Paraburkholderia* and *Burkholderia* into two distinct monophyletic clades and was strongly supported with very high bootstrap values (Figure 2B). This provides further phylogenomic evidence for the separation of these two genera (Figure 2).

**FIGURE 3:**
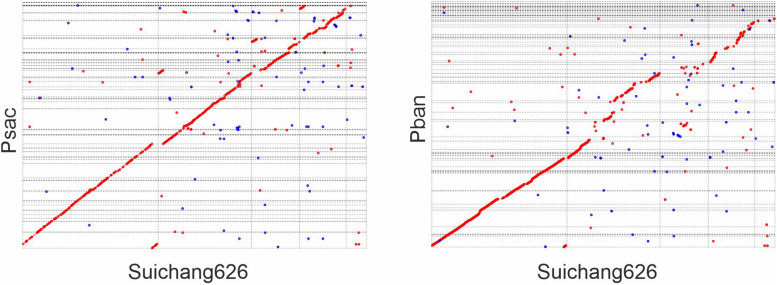
Dot-plot of whole genome alignment between *Paraburkholderia sacchari* Suichang626 and *P. sacchari* LMG19450 (Psac), and between *P. sacchari* Suichang626 and *P. bannensis* (Pban). Mummer 4.0.0beta2 (nucmer) was used for genome assembly comparison. Forward alignment was ploted in red and reverse as blue. Optimal co-linear order of contigs was shaped with mummerplot with parameter –fat. Mummerplot output.ps files were viewed and edited in CorelDRAW.

In order to determine if Suichang626 represents a novel species of *Paraburkholderia* or a strain of *P. sacchari* we calculated the following quantitative phylogenetic metrics with genomic data from all species examined here; average nucleotide identity (ANI), average amino acid identity (AAI), digital DNA-DNA hybridization (dDDH), and the tetranucleotide frequency correlation coefficient (TETRA). Comparisons of the Suichang626 and *P. sacchari* genomes supported that they are conspecific for three of these metrics (Tables 2, 3). Specifically, ANI was 96% [≥95–96% is conspecific (Richter and Rosselló-Móra, 2009)], AAI was 96% [≥95–96% is conspecific (Konstantinidis and Tiedje, 2005)], and TETRA was 0.99939% [>0.99 is conspecific (Richter and Rosselló-Móra, 2009)]. Exceptionally, dDDH did not support conspecificity with a value of 68%, which is close to but under the accepted species delineation border of ≥70% (Colston et al., 2014). Whole genome alignments between Suichang626 and *P. sacchari* exhibited a high level of conserved gene-synteny, further supporting that Suichang626 is a novel strain of the species *P. sacchari* (Figure 3). Finally, *B. mallei* and *B. pseudomallei* exhibited extremely high ANI, AAI, and dDDH scores, of 99%, 99%, and 93%, respectively (Table 2), suggesting their relationship may need to be reconsidered.

**TABLE 2:**
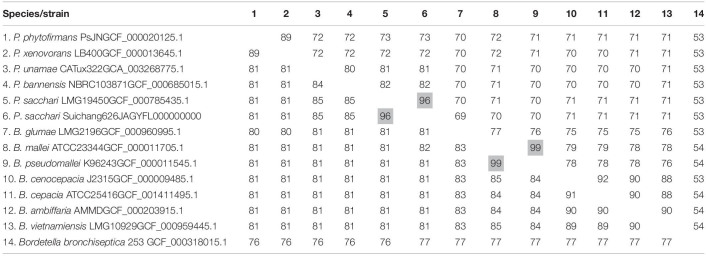
Average amino acid identity (AAI) and average nucleotide identity (ANI) values of selected *Paraburkholderia* and *Burkholderia* genomes.

*ANI (%) values are indicated in the lower triangle and AAI (%) in the upper triangle. Bordetella bronchiseptica has been included as an outgroup. Values above or close to bacterial species delineation threshold are highlighted with grey shade.*

**TABLE 3:**
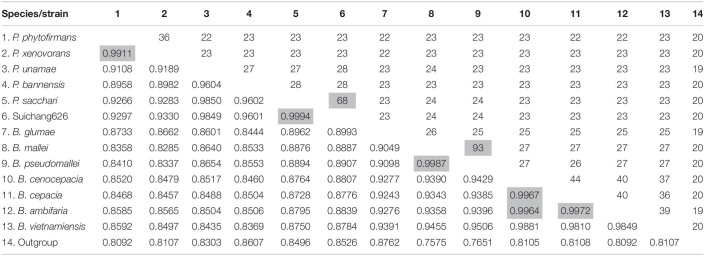
The digital DNA-DNA hybridization (dDDH) values and tetranucleotide frequency correlation coefficient (TETRA) of selected *Paraburkholderia* and *Burkholderia* genomes.

*TETRA are indicated in the lower triangle and dDDH (%) in the upper triangle. Bordetella bronchiseptica has been included as an outgroup for comparison. Suichang626: P. sacchari Suichang626. Values above or close to bacterial species delineation threshold are highlighted with grey shade.*

### Plant Growth-Promotion Genes in Suichang626

In order to gain insight into the mechanisms underlying the PGP effects of Suichang626, we explored the genes/proteins responsible for the biosynthesis of VOCs and phytohormones, as well as nitrogen fixation. Comparative genomic analysis was conducted with the genomes of 13 *Paraburkholderia* and *Burkholderia* species, representing both beneficial and plant/animal pathogenic species (Table 1).

The genome of Suichang626 contained full acetoin and 2,3-butanediol biosynthesis pathways, with genes encoding the key required enzymes (acetoin dehydrogenase and acetoin reductase; Table 4). The acetoin and 2,3-butanediol biosynthesis pathway was also present in the genome of the PGP species, *P. unamae*. Interestingly, all growth-promoting *Paraburkholderia* and *Burkholderia* species had the genes for acetoin synthesis in their genomes. In contrast, none of pathogenic *Burkholderia* species contained the full biosynthesis pathway for these metabolites. There was no homolog for acetoin dehydrogenase gene in the genomes of all *Burkholderia* pathogenic species, except *B. cenocepacia* (Table 4).

**TABLE 4:** Key enzymes involved in biosynthesis of volatile organic compounds acetoin and 2,3-butanediol.

	$	$	$	$		$	$	$	¤	¤	¤	¤	¤
	Pphy	Pxen	Puna	Pban	Psac	Sui	Bamb	Bvie	Bglu	Bmal	Bpse	Bcen	Bcep
Pyruvate decarboxylase	1	1	1	1	1	1	1	1	1	1	1	1	1
Acetolactate synthase	3	7	3	4	4	4	3	3	3	2	3	3	4
Acetoin dehydrogenase	1	1	2	1	1	3	1	1	–	–	–	1	–
Acetoin reductase	–	–	1	–	–	1	–	–	–	–	–	–	–

*Enzyme sequences from genome annotation (swissport) of Paraburkholderia sacchari Suichang626 were used as references. BLASTp was conducted against all annotations. Selection criteria are e-value < 1e-50, and similarity >40%. Protein sequence of acetolactate decarboxylase (Paraburkholderia sp. DHOC27 WP117337522.1) was searched and no hit was found from all tested species. Pphy, P. phytofirmans; Pxen, P. xenovorans; Puna, P. unamae; Pban, P. bannensis; Psac, P. sacchari; Sui, P. sacchari Suichang626; Bamb, B. ambifaria; Bvie, B. vietnamiensis; Bglu, B. glumae; Bmal, B. mallei; Bpse, B. pseudomallei; Bcen, B. cenocepacia; Bcep, B. cepacia. §, plant beneficial bacteria; ¤, plant/animal pathogen.*

Biosynthesis pathways of the phytohormones auxin and cytokinin were examined. In this study, we confirmed two species *P. xenovorans* and *B. vietnamiensis* had an intact synthesis pathway for the major auxin species, indole acetic acid (IAA). *P. xenovorans* had both the indole-3-acetamide and indole-3-pyruvic acid pathways, with one tryptophan 2-monooxygenases (TMO) and one indole acetamide hydrolase (IAMH) homolog for the indole-3-acetamide pathway; and two tryptophan aminotransferases (TAM), one indole-3-pyruvate decarboxylase (IPDC) and eleven indole-3-acetaldehyde dehydrogenase (IAD) homologs for the indole-3-pyruvic acid pathway (Table 5). *B. vietnamiensis* had only the indole-3-acetamide pathway, with one TMO and one IAMH homolog (Table 5). Other investigated species, including Suichang626, had no intact homologs for the characterized indole-3-acetamide or indole-3-pyruvic acid pathways. The key enzyme for cytokinin biosynthesis, isopentenyl transferase, were not found. However, a homolog of phosphoribohydrolase, an enzyme that transforms cytokinin nucleotides into active free-base form, was discovered in all species studied (Table 5).

**TABLE 5:** Homologs of bacterial plant hormone biosynthesis and modification genes.

Species	IAA IAM pathway		IAA IPyA pathway			Cytokinin		ACC deaminase
	TMO	IAMH	TAM	IPDC*	IAD	IPT	LOG	
*P. phytofirmans*	–	2	2	–/4	8	–	1	1
*P. xenovorans*	1	1	2	1/4	11	–	1	1
*P. unamae*	–	1	2	–/2	13	–	1	2
*P. bannensis*	–	1	1	–/2	12	–	1	1
*P. sacchari* LMG19450	–	1	2	–/3	5	–	1	1
*P. sacchari* Suichang626	–	1	2	–/3	8	–	1	–
*B. glumae*	–	–	3	–/1	4	–	1	1
*B. mallei*	–	1	2	–/2	4	–	1	2
*B. pseudomallei*	–	1	2	–/2	5	–	1	2
*B. cenocepacia*	–	–	2	–/2	6	–	1	1
*B. cepacia*	–	2	2	–/2	9	–	1	1
*B. ambifaria*	–	–	2	–/2	10	–	1	1
*Bordetella vietnamiensis*	1	1	2	–/2	4	–	1	1

Biosynthesis enzymes for the auxin – indole acetic acid (IAA), cytokinin biosynthesis enzymes, and ACC deaminase gene in Paraburkholderia and Burkholderia species genomes were presented. The accession numbers of primary and second query sequences are listed in section “Materials and Methods.” Selection criteria was e-value < e-70. *, number of IPDC homologs when the selection criteria was e < 1e-10. These putative IPDC have only 20–30% sequence similarity to the query. TMO, tryptophan 2-monooxygenase; IAMH, Indole acetamide hydrolase; TAM, tryptophan aminotransferase; IPDC, indole-3-pyruvate decarboxylase; IAD, indole-3-acetaldehyde dehydrogenase; ACC, 1-amino cyclopropane-1-carboxylate; IPT, isopentenyl transferase. LOG, cytokinin riboside 5′-monophosphate phosphoribohydrolase LOG. –, no hit. Bordetella bronchiseptica has been included as an outgroup for comparison.

**FIGURE 4:**
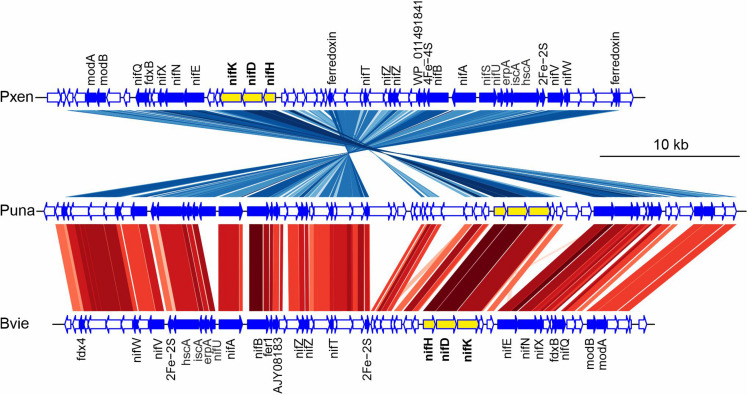
Gene synteny of nitrogen fixation gene clusters in *Paraburkholderia xenovorans* (Pxen), *P. unamea* (Puna), and *Burkholderia vietnamiensis* (Bvie). The genome region with nitrogen fixation gene cluster was analyzed as dnaseg in genoplotr package in R. Protein sequence comparisons by tBLASTx with the criteria, *e*-value < e-100 and bit score ≥100 were applied as comparison in genoplotr. Key to colored lines in gene comparisons: Darker shaded lines indicates strength of similarity, red indicates genes in the same orientation and blue reverse orientation. Key to gene colors: yellow, key nitrogen fixation cluster gene; blue, genes directly related to nitrogenase maturation and function; white, other putative genes. *fdx4*, ferredoxin gene; *nifW*, nitrogenase-stabilizing/protective protein gene; *nifV*, homocitrate synthase gene; *hscA*, Fe-S protein assembly chaperone gene; *iscA*, Fe-S cluster assembly protein gene; *erpA*, Fe-S cluster insertion protein gene; *nifU*, Fe-S cluster assembly scaffold gene; *nifS*, cysteine desulfurase gene; *nifA*, nif-specific regulatory protein gene; *nifB*, nitrogenase cofactor biosynthesis protein gene; *fer1*, ferredoxin-1 gene; *WP_011491841*, Fe-S cluster assembly accessory family protein gene; *AJY08183*, Fe-S cluster assembly accessory family protein gene; *nifZ*, nifZ domain protein gene; *nifT*, putative nitrogen fixation protein gene; *2Fe-2S*, Fe-S cluster binding domain protein gene; *4Fe-4S*, 4Fe-4S dicluster domain-containing protein gene; *nifH*, nitrogenase iron protein gene; *nifD*, nitrogenase molybdenum-iron protein alpha chain gene; *nifK*, nitrogenase molybdenum-iron protein beta chain gene; *knife*, nitrogenase MoFe cofactor biosynthesis protein gene; *nifN*, nitrogenase molybdenum-iron cofactor biosynthesis protein gene; *nifX*, nitrogen fixation protein gene; *fdxN*, ferredoxin III nif-specific gene; *nifQ*, nifQ family protein gene; *modB*, molybdate ABC transporter permease gene; *modA*, molybdate ABC transporter periplasmic molybdate-binding protein gene.

Suichang626 had no significant hit for the ACC deaminase gene in its genome (Table 5), suggesting that the growth-beneficial effect by Suichang626 is independent of this activity. However, the genomes of all other *Paraburkholderia* and *Burkholderia* species investigated harbored one or two homologs of ACC deaminase. The genome of Suichang626 lacked the nitrogenase reductase (*nifH*), FeMo dinitrogenase alpha (*nifD*), and beta (*nifK*) subunit genes, strongly suggesting that Suichang626 is unable to fix nitrogen for plants. Consistently, *nifHDK* genes were not present in the genome of *P. sacchari*, the species most closely related to Suichang626. Genomes of three other plant-beneficial species, *P. xenovorans*, *P. unamae* and *B. vietnamiensis*, contained a *nifHDK* gene cluster, as *nifHDK* homologs with significant similarity were annotated in their genomes and further confirmed with tBLASTn searches of their genome assemblies (Supplementary Table 1 and Figure 4). Additional nitrogen fixation accessory genes (*nif*) were also present in the genomes of *P. xenovorans*, *P. unamae*, and *B. vietnamiensis* with slightly different configurations (Figure 4). Moreover, synteny analysis of *nif* genes in *P. xenovorans*, *P. unamae*, and *B. vietnamiensis* demonstrates that all nif genes were located in a 40–50 kb island, indicating that the genes of nitrogenase biosynthesis, maturation, and function are tightly clustered. Secondary metabolite gene clusters were abundant and variable, in both the pathogenic and beneficial groups. Terpene, non-ribosomal peptide synthase, and bacteriocin gene clusters were present in all 13 *Paraburkholderia* and *Burkholderia* species (Supplementary Table 2).

## Discussion

### A Moso Bamboo Associated Strain of *Paraburkholderia sacchari*

We identified Suichang626 as a strain of *P. sacchari* with whole genome data (Figure 2 and Table 2). The many differences in gene content observed between the genomes of Suichang626 and the other strain of *P. sacchari* examined here indicates Suichang626 is a novel strain. ANI is strongly correlated with experimentally determined DNA-DNA hybridization values, thus has emerged as the new standard for bacterial species delimitation, especially when ANI and TETRA values agree (Richter and Rosselló-Móra, 2009). Based on the strength of the ANI and TETRA data presented above, we conclude that Suichang626 represents a strain of *P. sacchari*.

The mechanisms of PGP effects of microbes have been well-defined in other species (Sharma and Johri, 2003; Khalid et al., 2004; Kuklinsky-Sobral et al., 2004; Onofre-Lemus et al., 2009; Ambrosini et al., 2012; Nutaratat et al., 2014; Paungfoo-Lonhienne et al., 2014; Kurepin et al., 2015; Fu et al., 2016; Afzal et al., 2017; Kaur et al., 2017). Although several *Paraburkholderia* species with PGP effects were reported (Estrada-De Los Santos et al., 2001; Paungfoo-Lonhienne et al., 2014; Kaur et al., 2017), none of them are moso bamboo associated and PGP effects have not been previously observed in *P. sacchari*. Moso bamboo exhibits many traits that are desired for agriculture, such as fast growth, a perennial lifestyle, a large monocot growth habit, and stress tolerance. The use of microbial isolates from moso bamboo in crop promotion is attracting increased attention. Here, we isolated the *P. sacchari* strain Suichang626, which exhibited PGP effects for seedlings of both monocots and dicots (Figure 1). Here we have provided a high quality genome sequence of the *P. sacchari* strain Suichang626. The genome characteristics of Suichang626, such as genome size and GC content, fall within the criteria that distinguishes between the genus *Paraburkholderia* and *Burkholderia* (Sawana et al., 2014; Kaur et al., 2016; Mannaa et al., 2019). This genome information could serve as a reference resource for further studies on bamboo associated *Paraburkholderia* and potentially for biotechnological applications of *P. sacchari* in polyhydroxyalkanoate production, a biological alternative to petrochemical plastic that is produced with other strains of *P. sacchari* (Mendonça et al., 2014). Additionally, the genome-wide phylogeny analysis presented here supports the proposal that the genus *Paraburkholderia* is phylogenetically distinct from *Burkholderia* (Sawana et al., 2014; Kaur et al., 2017).

### Comparative Genomics of Plant Growth Promotion Genes

Acetoin and 2,3-butanediol are two key VOCs responsible for PGP effects, which have been identified from *Bacilli* strains interacting with *Arabidopsis thaliana* (Ryu et al., 2003; Farag et al., 2006). Full acetoin and 2,3-butanediol biosynthesis pathways were present only in the genomes of two plant beneficial bacteria, Suichang626 and *P. unamea* CATux322. No homologs of the acetoin reductase gene were found in the genomes of the 5 pathogenic *Burkholderia* species, suggesting that biosynthesis of acetoin and 2,3-butanediol is a unique trait of plant-beneficial bacteria (Farag et al., 2006).

The biosynthesis of the plant growth hormone auxin is a widespread trait of plant-associated microbes and of great importance in microbial-plant interactions (Spaepen et al., 2007; Zúñiga et al., 2013; McClerklin et al., 2018). The indole-3-acetamide pathway and indole-3-pyruvic acid pathways are the most common mechanisms of IAA synthesis in bacteria (Li et al., 2018; McClerklin et al., 2018). We identified the indole-3-acetamide pathway in the genomes of two PGP bacteria, *P. xenovorans* and *B. vietnamiensis*, but not in pathogenic *Burkholderia* species. However, since *ipdC* genes are difficult to identify from bacterial genomes (Li et al., 2018; McClerklin et al., 2018), species in the genera *Paraburkholderia* and *Burkholderia* may possibly utilize an alternate enzyme, IPDC, to complete IAA biosynthesis via the indole-3-pyruvic acid pathway. When using lower standard criteria in the secondary query, a group of IPDC homologs with 20-30% protein sequence similarity to IPDC of *P. xenovorans* were found in the Suichang626 genome (Table 5). These homologs are putative IPDC enzymes, which can be utilized for further experimental validation. Cytokinin is another plant hormone, frequently produced by microbes (Jameson, 2000; Pertry et al., 2010). *Paraburkholderia* and *Burkholderia* species may not have the capacity for *de novo* cytokinin production, as evidenced by the absence of isopentenyl transferase genes in their genomes, in spite of extensive searches. Nevertheless, some strains may interfere with their host plant’s endogenous cytokinin network to modify plant growth (Kurepin et al., 2015), possibly through cytokinin related enzymes such as phosphoribohydrolase LOG family proteins (Frébort et al., 2011). Many plant beneficial bacteria have ACC deaminase genes able to reduce ethylene levels in plants via breaking down the direct precursor to ethylene, ACC into ammonia and α-ketobutyrate, which can be utilized as microbial nutrients. This also releases the growth inhibition caused by ethylene production, a common plant-stress response (Glick et al., 1998; Govindasamy et al., 2008; Onofre-Lemus et al., 2009). For instance, an ACC deaminase gene in *B. phytofirmans* PsJN has major role on root elongation effect in canola seedlings (Sun et al., 2009). The presence of an ACC deaminase gene in a bacterial genome suggests the potential for being plant growth beneficial. However, genomes of 5 pathogenic *Burkholderia* species also contained ACC deaminase genes. This confirms that production of ACC deaminase is not a unique trait for PGP bacteria, but also a character for non-PGP bacteria (Belimov et al., 2007; Onofre-Lemus et al., 2009; Nascimento et al., 2014).

The ability to fix nitrogen is a widespread nutrient-oriented strategy in PGP bacteria, not limited to the *Rhizobia* that forms root nodules (Estrada-De Los Santos et al., 2001; Boyd and Peters, 2013). *Nif* genes were found in plant-beneficial species, but not in pathogenic *Burkholderia* species. *Nif* genes are typically found organized in clusters in the genomes of multiple bacterial taxa (Boyd and Peters, 2013). We have found that nitrogen fixation genes were clustered in a 50-kb region in the genera *Paraburkholderia* and *Burkholderia*. The conserved organization of these clusters suggests that the gain of nitrogen fixation ability in these two genera was possibly achieved by horizontal gene transfer. Key nif genes (*nifHDK*) have been found in a *Burkholderia* strain and *B. vietnamiensis* (Estrada-De Los Santos et al., 2001; Minerdi et al., 2001). Gene cluster content and synteny are conserved in this bacterial clade. *NifHDK* are highly conserved in all three species, and other accessory *nif* genes and ferredoxin genes are located nearby with conserved synteny. Exceptionally, *P. xenovorans*, and *B. vietnamiensis* have lost several genes in the cluster compared to *P. unamae* (Figure 4). *B. vietnamiensis* has lost the *nifS* gene, which encodes a cysteine desulfurase that is necessary for full activity of nitrogenase in *Azotobacter vinelandii* (Jacobson et al., 1989; Mihara and Esaki, 2002). The identification of the full *nif* gene cluster will help us to understand *nif* gene evolution and to further analyze the function of individual genes in the process of nitrogen fixation, by the experimental mutation or introduction of *nif* genes.

An overview of PGP mechanisms by *Paraburkholderia* and *Burkholderia* species is provided from the comparative genomic analysis presented here. PGP bacteria species, even within the same genus, apply different mechanisms to promote plant growth. Notably, the novel Suichang626 strain of *P. sacchari* can induce PGP on moso bamboo and *Arabidopsis*, the mechanism of which may involve the biosynthesis of acetoin and 2,3-butanediol. However, other known mechanisms were not addressed here (Glick, 2012; Olanrewaju et al., 2017), or yet uncharacterized mechanisms for growth promotion might also be involved. Importantly, the potential traits discussed here are purely based on genomic content. To gain a deeper understanding of PGP by Suichang626, further studies of the expression of these genes, how their enzyme activities are regulated, and functional studies of knockout mutants in PGP assays will be required. Moreover, how beneficial microbes survive in dynamic microbial communities in natural environments also requires further investigation, which will ultimately allow us to find biological crop management applications for sustainable agriculture.

## Data Availability Statement

The datasets presented in this study can be found in online repositories. The names of the repository/repositories and accession number(s) can be found below: https://www.ncbi.nlm.nih.gov/, Biosample: SAMN10358271 Bioproject: PRJNA503302 16S rRNA: MW741820.

## Author Contributions

FC, KW, and KO designed the experiments and conceived the data. KW performed the analysis and wrote the manuscript with FC and KO. YW, MY, and YY isolated the bacterial and performed plant treatments. YW prepare the samples for sequencing. All authors edited and approved the manuscript.

## Conflict of Interest

The authors declare that the research was conducted in the absence of any commercial or financial relationships that could be construed as a potential conflict of interest.

## Footnotes

1 https://blast.ncbi.nlm.nih.gov/

2 www.illumina.com

3 www.covaris.com
