# Supplementary material for: Comparative Genomics Reveals Potential Mechanisms of Plant Beneficial Effects of a Novel Bamboo-Endophytic Bacterial Isolate Paraburkholderia sacchari Suichang626
Source: Front Microbiol. 2021 Jun 18;12:686998. doi: 10.3389/fmicb.2021.686998 (PMC8250432; doi:10.3389/fmicb.2021.686998)
Supplement: Supplementary file 2 [file Data_Sheet_2.pdf]

Supplementary Table 1. Homologs of nitrogen fixation genes in the genome of *Paraburkholderia* and *Burkholderia* species, including *P*. sp. Suichang626 (Sui). $: Plant beneficial bacteria; ¤: plant/animal pathogenic bacteria; -: No hit.

	$	$	$	$		$	$	$	¤	¤	¤	¤	¤
	Pphy	Pxen	Puna	Pban	Psac	**Sui**	Bamb	Bvie	Bglu	Bmal	Bpse	Bcen	Bcep
nifH	-	1	1	-	-	-	-	1	-	-	-	-	-
nifK	-	1	1	-	-	-	-	1	-	-	-	-	-
nifD	-	2	2	-	-	-	-	2	-	-	-	-	-
4Fe-4S	2	2	4	3	3	3	2	3	2	2	2	3	2
2Fe-2S	1	2	2	1	1	-	1	2	1	1	1	1	1

Supplementary Table 2. Secondary metabolite gene clusters in the genome of *Paraburkholderia* and *Burkholderia* species, including *P*. sp. Suichang626 (Sui). Siderophore of Puna moved to NRPs. $: Plant beneficial bacteria; ¤: plant/animal pathogenic bacteria; -: No hit.

	$	$	$	$		$	$	$	¤	¤	¤	¤	¤
	Pphy	Pxen	Puna	Pban	Psac	**Sui**	Bamb	Bvie	Bglu	Bmal	Bpse	Bcen	Bcep
Terpene	3	3	5	4	5	5	4	3	3	4	4	5	6
NRPs	2	1	1	3	2	2	1	2	**7**	**7**	11	3	3
PKs	-	-	-	3	1	1	1	2	**7**	**7**	8	1	1
Lassopeptide	-	-	-	-	-	-	-	-	-	1	1	-	-
Bacteriocin	2	2	3	4	3	3	1	1	2	2	3	1	1
Phosphonate	2	1	1	-	1	1	1	1	1	1	1	1	1
Hserlactone	2	1	1	-	1	2	1	3	1	2	3	1	1
Ladderane	-	-	-	-	1	1	-	-	-	-	-	-	-
Batalactone	-	-	-	1	-	1	1	-	1	-	1	1	-
Arylpolyene	-	1	1	1	-	1	2	2	1	-	-	1	1
Phenazine	-	-	-	-	-	-	-	-	1	-	-	-	-
